# Blood Requisition and Utilization Practice in Surgical Patients at University of Gondar Hospital, Northwest Ethiopia

**DOI:** 10.1155/2013/758910

**Published:** 2013-11-28

**Authors:** Tadesse Belayneh, Gashaw Messele, Zewditu Abdissa, Birehanemeskel Tegene

**Affiliations:** ^1^Department of Medical Anesthesiology, College of Medicine and Health Sciences, University of Gondar, Kebele 16, P.O. Box 196, Gondar, Ethiopia; ^2^Department of Surgery, College of Medicine and Health Sciences, University of Gondar, Kebele 16, P.O. Box 196, Gondar, Ethiopia; ^3^Department of Medical Microbiology, College of Medicine and Health Sciences, University of Gondar, Kebele 16, P.O. Box 196, Gondar, Ethiopia

## Abstract

*Background.* Although blood ordering is a common practice in surgical field, the average requirement for a particular procedure is usually based on subjective anticipation of blood loss rather than on evidence based estimates. Overordering with minimal utilization squanders technical time, reagent and imposes extra expenses on patients. This study was conducted to assess blood utilization practices. *Methods*. Cross-sectional study was conducted in Gondar Hospital. Five-month data were collected from all discharged surgical patients and blood bank registries. Blood utilization was calculated using crossmatch to transfusion ratio (C/T), transfusion probability (%T), and transfusion index (TI) indices. *Results.* A total of 982 patients were requested to prepare 1,072 crossmatched units. Of these, 468 units were transfused for 286 patients. The overall ratios of C/T, %T, and TI index were 2.3, 47%, and 0.77, respectively. Blood transfusion from the units crossmatched was 43.6%. Moreover, the highest C/T ratio was observed in elective surgical patients. *Conclusions.* The overall blood utilization was encouraging, but excessive crossmatching with minimal transfusion practice was observed in elective surgical patients. Blood ordering pattern for elective procedures needs to be revised and overordering of blood should be minimized. Moreover, the hospital with blood transfusion committee should formulate maximum surgical blood ordering policies for elective surgical procedures and conduct regular auditing.

## 1. Introduction 

Blood transfusions play a major role in the resuscitation and management of surgical patients [1] and ordering of blood is usually a common practice in elective and emergency surgical procedures [2].

The preoperative request of blood units, especially in elective surgery, is often based on the worst case assumptions, demanding large quantities of blood or overestimating the anticipated blood loss, of which little is ultimately used [1]. This may cause exhaustion of valuable supplies and resources both in technician time, effort, and biochemical reagents. It also adds to financial burden for each patient undergoing a surgical procedure [3]. Increasing demand for blood and blood products together with rising cost and transfusion associated morbidity led to a number of studies that review blood ordering and transfusion practice [4, 5].

Since the introduction of blood transfusion into clinical practice, its appropriate use has been the subject for debate. It has been reported that only 30% of crossmatched blood is used in elective surgery [6].

In addition, a number of studies in many countries of the world have shown overordering of blood by surgeons with utilization ranging from 5 to 40% [4]. In South Africa, for example, 7–10% of blood is wasted annually because of overordering of blood [7]. Reports from India, Kuwait, and Nigeria also showed utilization rate of 28% [1], 13.6% [8], and 69.7% [3], respectively. Even in trauma patients, utilization is less than 50% [9]. This study has also shown excessive cross matching of blood for elective surgeries with only 30.0% of blood utilized.

Common variations in rates of transfusion may be due to many factors, including differing opinions on the threshold level of hemoglobin below which a patient needs blood transfusion, differences in surgical and anesthetic techniques, cancellation of cases, differences in case mix, preoperative anemia, and lack of availability of transfusion protocols. This may reflect uncertainty about the relative benefits and risks of transfusion and the different perceptions of the value of minimizing blood loss and subsequent transfusion [10].

A number of indices are used to determine the efficiency of blood ordering and utilization system. Boral Henry was the first that suggested the use of crossmatch to transfusion ratio (C/T ratio) in 1975 [4]. Consequently, a number of authors used C/T ratio for evaluating blood transfusion practice. Ideally, this ratio should be 1.0, but a ratio of 2.5 and below was suggested to be indicative of efficient blood usage [3]. The probability of a transfusion for a given procedure is denoted by %T and was suggested by Mead et al. in 1980 [11]. A value of 30% and above has been suggested as appropriate [3]. The average number of units used per patient crossmatch is indicated by the transfusion index (TI) and signifies the appropriateness of number of units crossmatched. A value of 0.5 or more is indicative of efficient blood usage [3, 4].

Unnecessary ordering of blood for surgical patients can be reduced without having any detrimental effect on the quality of patient. Use of blood conservation policies such as the MSBOS has succeeded in limiting unnecessary transfusion practices [12]. Maximal Surgical Blood Order Schedule (MSBOS = 1.5 × TI) estimates the amount of blood that will be needed for the individual procedure. This is a criterion developed from institutional usage statistics providing a figure for the number of units to be crossmatched for any given surgical procedure [13]. In the surgeries which have insignificant blood loss, only blood grouping of the patient should be done and crossmatching can be avoided which can not only be rational and cost effective but also hasten the time lost in waiting for surgery. However, one must confirm the availability of blood for emergency situation before starting the surgery [4]. Many studies [1, 14] have shown that blood is generally over ordered and the implementation of MSBOS and the introduction of “T and S” procedure have led to a safe, effective, and economic solution to ordering of blood.

Evaluating blood ordering and transfusion practices and subsequent developing of a blood ordering schedule, which serves as a guide to anticipated normal blood usage for elective and emergency surgical procedures, can decrease overordering of blood. University of Gondar Hospital has no blood bank for its own, and it gets the necessary blood for its patients from Regional blood bank Branch of Ethiopian Red Cross Society. The Blood Bank has regular voluntary blood collection campaign schedule. But it does not fulfill the requirement of the need of patients in the hospital. Due to these, the patients are forced to collect their blood either from their relatives (family, friends) or paid donors. In this regard, studies assessing blood requisition and transfusion practices are scarce in Ethiopia particularly in Gondar Hospital. Therefore, the aim of this study was to assess the efficiency of blood requisition and transfusion practices for patients undergoing both elective and emergency surgical procedures.

## 2. Materials and Methods

A hospital based cross-sectional study was conducted in those patients who underwent elective and emergency surgeries in University of Gondar Hospital over a period of five-months from November 2012 to March 2013. It is a 5000-bed tertiary teaching hospital performing 11,238 surgical procedures per year.

Blood requisition and transfusion of surgical, obstetrics, and gynecological cases were compiled and reviewed. Patient's age and sex, number of units prepared, crossmatched, and transfused, number of patients crossmatched and transfused, source of blood donation or collection, and type of anesthesia were collected from discharged patient medical records and blood bank registries.

Data were coded, entered, and analyzed using SPSS Version 16. Blood utilization indices were computed with the following equation.

- Crossmatch to transfusion ratio (C/T ratio) = number of units crossmatched/number of units transfused. A ratio of 2.5 and below is considered indicative of significant blood usage.
- Transfusion probability (%T) = number of patients transfused/number of patients crossmatched × 100. A value of 30% and above was considered indicative of significant blood usage.
- Transfusion index (TI) = number of units transfused/number of patients crossmatched. A value of 0.5 or more was considered indicative of significant blood utilization.
- Maximal Surgical Blood Order Schedule (MSBOS) = 1.5 × TI.
- In current study blood was wasted when a patient failed to use his/her already prepared blood in any case.

Ethical clearance was taken from Ethical Board of University of Gondar. Participants were communicated individually about the purpose of the study and verbal informed consent was taken before the interview. Confidentiality of the information was assured by using code numbers than personal identification names and keeping questionnaires locked.

## 3. Results

During the study period a total of 1,412 patients underwent major elective and emergency surgical procedures. Among these, 982 patients were requested to prepare 1,072 units of blood.

Majority of the patients were female (51.9%), underwent surgery in elective schedule, surgical cases in surgical department, underwent surgery under general anesthesia and prepared their blood from replacement donation, as shown in Table 1. Patients prepared blood ranging from 1 unit to 3 units; averagely it was 1.09. From 468 units transfused, 291 (62.2%) units were transfused postoperatively.

## 4. Blood Requisition and Utilization in Respective Departments

During the five months of study period, blood requisition was made to 644 patients undergoing surgery in surgical department and 428 from gynecologic and obstetrics department (Gyn/obs). From a total of 1,072 units of blood crossmatched, only 468 units were transfused. These showed that 43.6% of total blood crossmatched was utilized, leaving 56.4% of the units crossmatched not transfused to the patient who prepared, that is, wasted.

Gynecology and obstetrics department was the department with the highest number of both patients crossmatched (63.2%) and transfused (64.4%). On the other hand, surgery was the department with the highest number of both blood units crossmatched (60.1%) and transfused (59.8%). Of the 354 patients who underwent emergency operation, 418 units of blood were crossmatched and 209 patients received 230 units of blood transfusion. In case of patients who underwent elective operation, 654 units of blood were crossmatched, out of which only 77 patients received 238 units of blood for their procedures. Generally the highest crossmatched units but the least transfusion units were made for elective patients as compared to emergency cases, as shown in Table 2.

## 5. Blood Utilization Indices of Operated Patients in the Respective Departments 

As shown in Table 3, generally the overall blood transfusion of the requested blood which was explained by indices of C/T ratio, %T, and TI were 2.3, 47%, and 0.77, respectively. These blood utilization indices showed a different value between each department.

In department of surgery, for instance, overall C/T ratio was 2.3 with high ratio in elective than emergency (2.6 versus 1.7). In addition, the overall %T was 46.2% with 35.0% in emergency and 79.3% in elective patients.

On the other hand, in department of gynecology and obstetrics, the overall C/T ratio was 2.3 with elective surgical patients having the highest C/T ratio (2.9) than emergency patients (1.8). Moreover, the overall %T and TI were 48.2% and 0.49, respectively.

## 6. Discussion

Blood and its component play a major role in the resuscitation and management of both elective and emergency surgical patients. Despite this advantage, currently there is a limited supply with increasing demand and underutilization of the requested blood worldwide [3].

Preoperative overordering of blood has been documented since 1976, when Friedman et al. published their findings. Subsequently, a number of studies have also showed over ordering of blood in different parts of the countries [1, 4]. Data from developing countries have shown gross over ordering of blood in 40% to 70% of patients transfused [8].

Since the introduction of blood transfusion into clinical practice, its appropriate use has been the subject of debate. It has been reported that only 30% of crossmatched blood is used in elective surgery [6, 15]. Generally the percentage of crossmatched patients receiving transfusion for general surgical procedures ranged from 5 to 40% [3]. Therefore, it is essential that the usage of blood and blood products should be rationalized and saved for crisis situations.

The current study revealed that 56.4% of the cross- matched blood was unutilized. This finding was almost comparable to that reported in northern India study where 59.0% of blood crossmatched was unutilized [16]. But it was relatively low compared to a study conducted in India (76.8%), Nigeria (69.7%), Nepal (86.4%), and Egypt (74.8%) [1, 3, 8, 17]. This might indicate that this malpractice is common in developing countries including our Ethiopia.

Boral Henry was the first, and a number of authors then after, used crossmatch to transfusion ratio [4] for evaluating blood transfusion practices. Ideally, this ratio should be 1.0, but a ratio of 2.5 and below was suggested to be indicative of efficient blood usage. According to this recommendation, the overall C/T ratio of 2.3 that was reported in current study was considered to be indicative of efficient blood usage. This ratio was comparable with that reported by a study conducted in Nigeria (2.2) [3] and Indian (2.5) [18] but lower than that reported by a study conducted in Egypt (3.9) [17] and Malaysia (5.0) [19].

The present study also demonstrated that C/T ratio was similar across the emergency patients of surgical (1.7) and gynecology and obstetrics (1.8) departments. This was somewhat similar to that reported in Egyptian [17] and Nigerian studies [3]. In contrast, C/T ratio was widely varied and high across elective patients of surgery (2.6) and gynecology and obstetrics (2.9) departments. This was similar with study conducted in northern India [18]. Disparities in rates of transfusion in the current study are due to the fact that there is a great tendency to request more units of blood for elective procedures than what is actually required in each department. This over ordering of blood might due to subjective over blood loss estimation of a procedure by surgeons which usually explain for the provision of safety measure in the event of excessive unexpected blood loss during surgery.

Mead et al. [11] suggested the probability of transfusion for a given procedure (%T), which indicates efficient use of blood. Accordingly, a value of 30% and above has been suggested to be appropriate and signifies the appropriateness of number of units crossmatched [11]. Based on what is recommended in the above literature, the results of the present study revealed an overall %T of 47.0%, which was indicative of appropriate utilization compared to unit crossmatched. This finding was higher than that which has been found in study conducted in Indian tertiary care hospital where %T ranged from 11.1% to 25% [1] and in Egypt where it was 36.9% [17]. Similarly, the probability of transfusion (%T) reported in different departments under the current study was considered appropriate except for elective patients in gynecology and obstetrics department (23.3%) which showed inefficient utilization.

Regarding transfusion index (TI), a value of 0.5 or more is indicative of efficient blood usage and signifies the appropriateness of number of units transfused [4]. The TI reported in the current study was 0.77. Transfusion index (TI) of elective and emergency patients under the study was considered appropriate in both departments except for emergency patients (0.44) in the department of gynecology and obstetrics. This finding was higher than that which has been found in a study conducted in Indian tertiary care hospital 0.36 [1] and Egypt in 0.69 [17]. Blood ordering pattern needs to be revised and overordering of blood should be minimized. This can be possible by the estimation of MSBOS for each procedure and requisition as calculated. Many studies [1, 14] have shown that blood is generally over ordered and the implementation of MSBOS and the introduction of “T and S” procedure have led to a safe, effective, and economic solution to ordering of blood.

In conclusion the overall ratio of C/T, %T, and TI index were considered to be optimal as compared with the standard figures, even though majority of the crossmatched blood was not utilized by the patient. In vast majority of elective surgical procedures routine crossmatch and preparation are not necessary. Moreover C/T ratios of elective patients in each department showed inefficient utilization of ordered blood. Developing a blood ordering policy, which is a guide to expect normal blood usage for surgical procedures, can decrease over ordering of blood thereby reducing unnecessary compatibility testing, returning of unused blood, and wastage due to outdating. It also allows for a more efficient management of blood inventory. In this respect, the hospital blood transfusion committee should formulate maximum surgical blood order schedules for selected cold surgical procedures, conduct regular auditing about effectiveness of the blood requesting policy using the crossmatch to transfusion ratio, and offer periodic feedbacks to improve blood ordering, handling, distribution, and utilization practices of this scarce resource.

## Figures and Tables

**Table 1:** Sociodemographic and other characteristics surgical patients at University of Gondar Hospital, Ethiopia, 2013 (*N* = 1,212).

S.N	Characteristics	Total (no.)	Percent (%)
1	Sex		
	Male	472	48.1%
	Female	510	51.9%
2	Source of donation		
	Voluntary	176	17.9%
	Replacement	896	82.1%
3	Type of operation		
	Elective	628	63.9%
	Emergency	354	36.1%
4	Type of anesthesia		
	General	668	68.0%
	Spinal	314	32.0%
5	Department/cases		
	Surgery	599	60.9%
	Gynecology and obstetrics	383	39.1%
6	Total units of blood prepared	1,072	100%
7	Total units of blood crossmatched	1,072	100%
8	Total units of blood transfused	468	43.6%
9	Preoperative blood units replaced	34	7.2%
10	Intraoperative blood units replaced	143	30.6%
11	Postoperative blood units replaced	291	62.2%
12	Total units of blood wasted	604	56.4%

**Table 2:** Comparison between number of units crossmatched and transfused in operated patients in University of Gondar Hospital, Ethiopia, 2013.

Department	Number of units		Number of patients	
	Crossmatched (*N* = 1072)	Transfused (*N* = 468)	Crossmatched (*N* = 602)	Transfused (*N* = 286)
Surgery (*N* = 599)				
Elective (*N* = 392)	412 (63.9)	154 (55.0)	54 (24.3)	43 (42.1)
Emergency (*N* = 207)	232 (36.1)	126 (45.0)	168 (75.7)	59 (57.9)
Subtotal	644 (60.1)	280 (59.8)	222 (36.8)	102 (35.6)
Gynecology and obstetrics (*N* = 383)				
Elective (*N* = 236)	242 (56.5)	84 (44.6)	146 (38.4)	34 (18.4)
Emergency (*N* = 147)	186 (43.5)	104 (55.4)	234 (61.6)	150 (81.6)
Subtotal	428 (39.9)	188 (40.2)	380 (63.2)	184 (64.4)

*N*: total number.

**Table 3:** Blood utilization indices of surgical patients at University of Gondar Hospital, Ethiopia, 2013 (*N* = 1,212).

Department	Blood utilization indices								
	C/T ratio			%T			TI		
	N	D	I	N	D	I	N	D	I
Surgery									
Elective (*N* = 392)	412	154	2.6	43	54	0.79	154	54	2.85
Emergency (*N* = 207)	232	136	1.7	59	168	0.35	136	168	0.81
Subtotal	644	280	2.3	102	222	0.46	280	222	1.26
Gynecology and obstetrics									
Elective (*N* = 236)	242	84	2.9	34	146	0.23	84	146	0.57
Emergency (*N* = 147)	186	104	1.8	150	234	0.64	104	234	0.44
Subtotal	428	188	2.3	184	380	0.48	188	380	0.49
Total	**1072**	**468**	**2.3**	**286**	**602**	**0.47**	**468**	**602**	**0.77**

Abbreviations: N stands for numerator; D stands for dominator; I stand for index. C/T: crossmatch transfusion ratio, %T: the probability of transfusion, and TI: transfusion index.
